# Delineation of an inverted tandem Xq23-26.3 duplication in a female featuring extremely short stature and mild mental deficiency

**DOI:** 10.1186/s13039-023-00663-z

**Published:** 2023-11-29

**Authors:** Shengfang Qin, Jiuzhi Zeng, Jin Wang, Mengling Ye, Qin Deng, Xueyan Wang, Zhuo Zhang, Dangying Yi, Yang Wu, Jesse Li-Ling

**Affiliations:** 1 Department of Medical Genetics and Prenatal Diagnosis, Sichuan Provincial Maternity and Child Health Care Hospital, Chengdu, 610045 Sichuan China; 2 West China Second University Hospital, Sichuan University, Chengdu, 610041 Sichuan China; 3 Department of Reproductive Medicine, Sichuan Provincial Maternity and Child Health Care Hospital, Chengdu, 610045 Sichuan China

**Keywords:** Short stature, Intellectual disability, Xq23-q26.3 duplication

## Abstract

**Background:**

Partial duplications involving the long arm of the X chromosome are associated with mental retardation, short stature, microcephaly, and a wide range of physical findings. Female carriers usually have no clinical phenotype. Occasionally, they may also have heterogeneous features due to non-random inactivation of the X chromosome.

**Methods:**

The peripheral blood sample was collected from the patient and subjected to a few genetic testing, including chromosomal karyotyping, Chromosomal microarray analysis (CMA), Optical genome mapping, short tandem repeat (STR) analysis for Determination of parental origin, and X chromosome inactivation (XCI) analysis.

**Results:**

We have identified a de novo Xq23-Xq26.3 duplication in an adult female featuring extremely short stature and mild mental deficiency. Chromosome analysis detected a duplication on Xq23-q26.3 with a size of approximately 20 Mb. The duplication region has encompassed a number of genes, among which *ARHGEF6*, *PHF6*, *HPRT1* and *SLC9A6* are associated with X-linked mental retardation. Further analysis suggested that the duplication has derived from her father, was of the inversion duplication type and involved various degrees of skewed X chromosome inactivation.

**Conclusion:**

Correlation with her phenotypes might indicate new mechanisms by which the X chromosome may lead to short stature and mental retardation. Our findings thereby may shed more light on the phenotypic implication of functional disomy of X-chromosome genes.

## Background

X chromosome accounts for approximately 5% of the human genome [1]. Compared with the autosomal chromosomes, genes underlying X-linked diseases are easier to identify because of their particular inheritance pattern. Generally speaking, female carriers usually have no clinical phenotype but may give birth to affected sons. Occasionally, they may also have mild features due to non-random inactivation of the X chromosome.

The X chromosome has been implicated in body height, intelligence, and fertility [2–5]. In addition to Turner syndrome (monosomy X), the *SHOX* gene mapped to the pseudo-autosomal region 1 (PAR1) at Xp22 has also been associated with body height. X-linked mental retardation (XLMR) is a group of highly heterogeneous disorders which affect 1.8/1000 males and 2.4/1000 females [6]. So far, over 100 XLMR genes have been identified [7], with the most common ones including MECP2 and FMR1 [8]. The associated mental retardation is divided into syndromic and non-syndromic types, with the latter having additional skeletal malformation and reproductive anomalies.

We report on an ethnic Chinese adult female featuring extremely short stature, facial dysmorphism, infertility, and mild mental deficiency. With combined genetic methods, the duplication fragments are explored in detail. Our finding may shed light on the mechanisms underlying the determination of body height, intelligence, and reproductivity.

## Methods

### Collection of specimen and DNA extraction

With informed consent obtained, peripheral venous blood samples were taken from the patient and her parents with tubes containing heparin sodium and EDTA-Na_2_ anticoagulants, respectively. Oral mucosal cells were collected from buccal smears, and urethral epithelial cells were isolated by centrifugation of the urine sample. By following the protocol provided by the manufacturer, genomic DNA was extracted from the specimen using the QIAamp DNA Mini Kit (QIAGEN, Germany). The DNA was qualified when the concentration was above 30 ng/uL, and the OD260/280 value was between 1.8 and 2.0, as determined with an ultraviolet spectrophotometer Nanodrop 1 C (Thermo Fisher Scientific, USA).

### Chromosomal karyotyping

Lymphocytes and amniocytes were cultured, harvested, and prepared for microscope slides before Giemsa staining adequately describes classical G-banding of metaphases. A karyotype analysis system (Karl Zeiss, Germany) was adopted for chromosome count and karyotype analysis.

### Chromosomal microarray analysis (CMA)

500 –1000 ng of genomic DNA was used for the CMA assay with a SurePrint G3 CGH + SNP (180 K) microarray chip. Potential CNVs were detected with Agilent CytoGenomics software and some online databases. The pathogenicity was judged based on the standards and guidelines from the American College of Medical Genetics and Genomics(ACMG) [9].

### Optical genome mapping

To delineate the chromosomal structural rearrangement, the DNA sample of the patient was further analyzed by whole-genome optical genomic mapping (OGM), an accurate and highly reproducible method for genome-wide SV analysis and delineation of complex genomic rearrangements [10]. The ultra high molecular weight DNA from the patient’s blood was isolated with the SP Blood and Cell Culture DNA Isolation Kit, and fluorescently labeled with the enzyme DLE-1 as per the manufacturer’s directions. Labeled DNA was loaded on Saphyr chip and imaged on the Saphyr instrument, for collection of 1300 Gb of molecules > 150 kb. De novo genome assemblies and variant calling were performed via Bionano Access software (v1.4.3) using the Bionano Tools version 1.4.3. The kit, the instrument, and the analysis software were provided by Bionano Genomics.

### Determination of parental origin

Short tandem repetition (STR) analysis was performed by multiplex fluorescence quantitative PCR amplification with a chromosome aneuploidy detection kit (Darui Corp., China). The amplification condition was 95 °C for 15 min, 94 °C for 30 s, 58 °C for 1 min 30 s, 72 °C for 1 min 30 s, for 27 cycles, and 72 °C for 30 min. The product was subjected to capillary electrophoresis with an AB 3500Dx gene analyzer, and the data were analyzed with GeneMapper software. The fluorescence peaks of the patient were compared with those of her parents.

### Analysis of X chromosome inactivation patterns

The DNA samples from the patient’s peripheral blood cells, oral mucosal cells, and urethral epithelial cells were amplified by androgen receptor (*AR*) gene-specific primers and subjected to capillary electrophoresis. The samples proceeded to the same tests after being digested with HpaII, a methylation-sensitive restriction enzyme. The XCI ratio was calculated with the formula (d1/u1)/(d1/u1 + d2/u2), and the skewed XCI was confirmed when the XCI ratio was > 70% [11].

## Results

### Case presentation

**Fig. 1:**
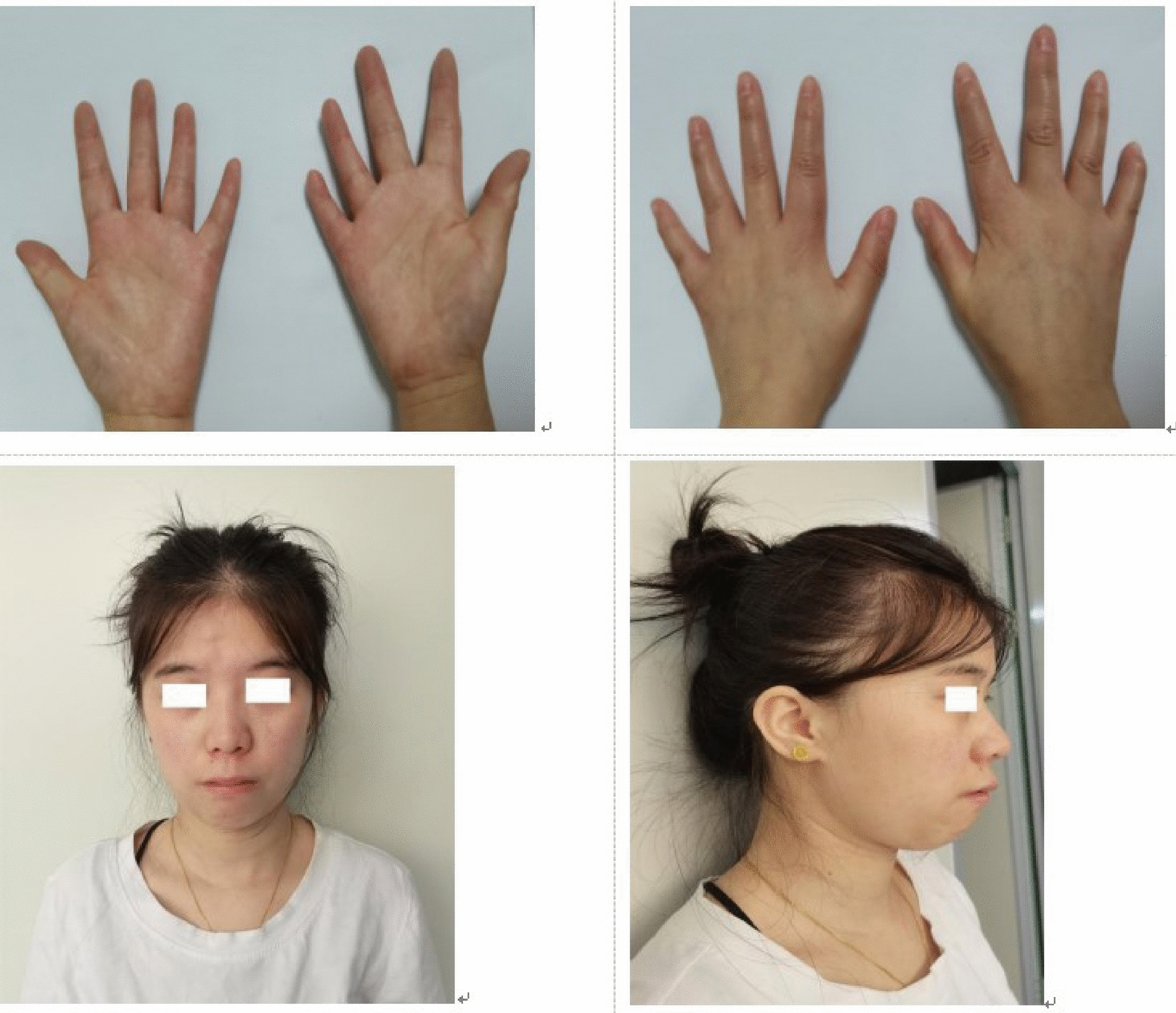
Clinical phenotypes of the patient the patient has tapering fingers, contracture of the distal joint of the right 5th finger, triangular face, and mild micrognathia

The patient, a 26-year-old female, has a height of 135.6 cm (< 2SD) and a weight of 32.15 kg (< 2SD). Physical examination revealed proportionate dwarfism. The patient was born at full term. Her parents had an average height. Her father was about 170 cm, and her mother was about 160 cm. The patient has had a poor appetite and slow eating since childhood. She was more irritable and bad-tempered, with poor muscle strength, learning difficulties (completed the nine-year compulsory education), and slightly better Chinese but poor math (non-verbal learning difficulties). Her height and weight had always lagged far behind her age, and this has aggreviated after age 7. She had menarche at around 15, with regular but reduced menstruation, and often had dysmenorrhea. She was married for two years but had not conceived without contraception. She had deformities such as small hands, tapered fingers, right fifth finger flexion, triangular face, slight hypertelorism, thin lips, and mild micrognathia (Fig. 1).

The patient had normal breast development. Ultrasonography of the abdomen revealed an anteverted uterus about 4.5 × 3.7 × 3.9 cm. The right uterine horn could be seen, but the left uterine horn was not shown, only a hypoechoic area about 3.0 × 1.0 cm in the left accessory area connected to the left wall of the uterus. Iohexol hysterosalpingography further revealed a unilateral uterus and incomplete obstruction of the right fallopian tube. The patient’s total score on the Hamilton Anxiety Scale was 5 (< 7) and did not hint at anxiety. The total score on the Hamilton Depression Scale was 17 (between 8 and 20), which indicated mild depressive symptoms. Laboratory test of endocrinology revealed progesterone (PRGE) at 0.33 ng/ml [reference value (RV): follicular phase 0.31–1.52); Estradiol (E2) 43 pg/ml (RV: 15.16-127.81 at the early follicular stage); Luteinizing hormone (hLH) 3.27 mIU/ml (RV: 2.12–10.89 in follicular phase); Follicle stimulating hormone (hFSH) 7.76 mIU/ml (RV: 3.85–8.78 at the follicular stage); Human chorionic gonadotropin (β-HCG) < 0.50 mIU/ml (RV: <0.5–2.90); Anti-Müllerian hormone (AMH) 2.56 ng/ml (RV: 0.96–13.34); Prolactin (RPL) 250.47 µIU/ml (RV: 71–566); Testosterone (TSTO) 25.55ng/dl (RV: 0–92). The patient’s parents were healthy and with a normal karyotype. They have denied consanguinity and a family history of genetic diseases.

All procedures in this study conformed to the tenets of the Helsinki Declaration and were approved by the Sichuan Provincial Maternity and Child Health Care Hospital institutional review board. Informed consent was obtained from all participants or guardians before collecting clinical data, venous blood, oral mucosa, and urethral epithelial cell samples.

### Cytogenetic analysis

**Fig. 2:**
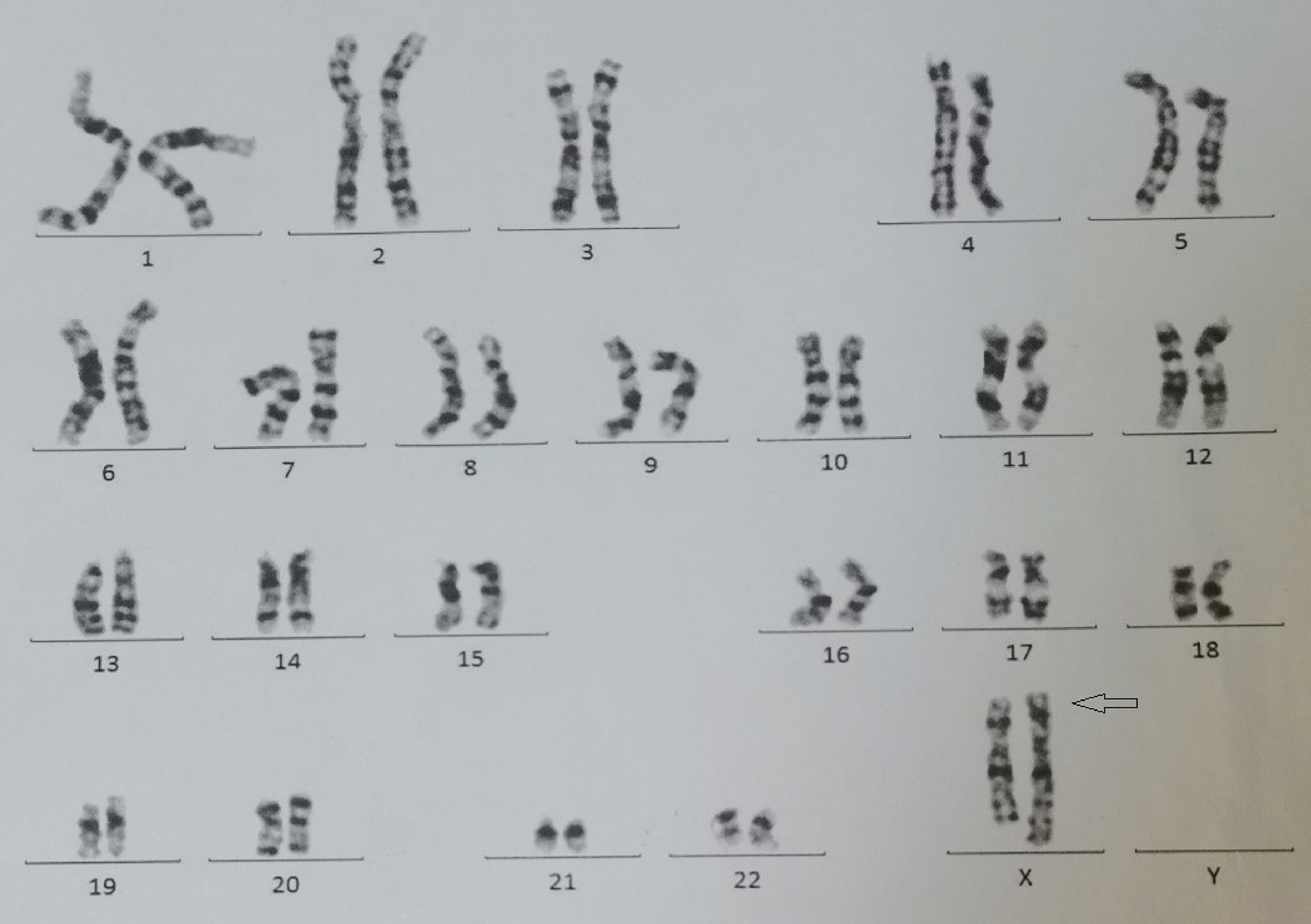
Karyogram of the patient the patient has a karyotype of 46,X,?der(X)(q28) with an apparent duplication on the long arm of one of the X chromosomes (indicated by the arrow)

G-banded chromosome analysis suggested that the patient has a karyotype of 46,X,?der(X)(q28) (Fig. 2). The duplication was found on the long arm of one of the X chromosomes.

### Chromosomal microarray analysis (CMA)

**Fig. 3:**
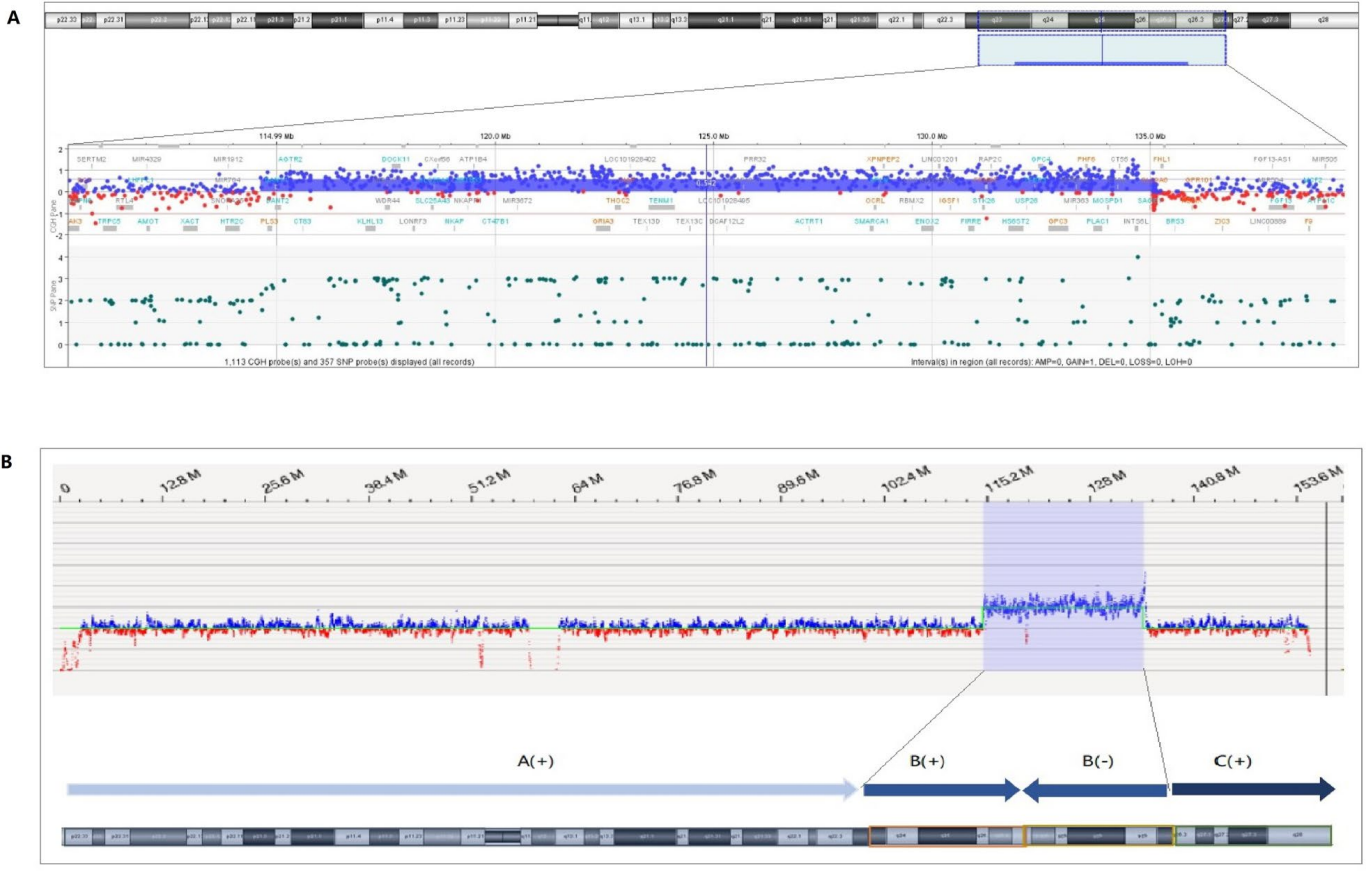
CMA and OGM results of the X chromosome ** A** CMA result of the X chromosome. The patient has a 20.45 Mb duplication at Xq23-q26.3 (hg19 chrX: 114,622,584–135,068,006) (represented by the blue bar) containing 121 protein-coding genes. **B** OGM result of the patient’s X chromosome. The resulting map of the X chromosome copy number showing a 19.9 Mb duplication (copy number = 3) at Xq23q26.3 (hg19 chrX: 114,624,594–134,525,997) (blue shaded area), which has encompassed 107 protein-coding genes). The repeat region at Xq23-q26.3 showed a pattern of reverse tandem repeats. Compared with the reference, the repeat sequence [B(−)]was in reverse tandem arrangement with the original sequence[B(+)]

**Fig. 4:**
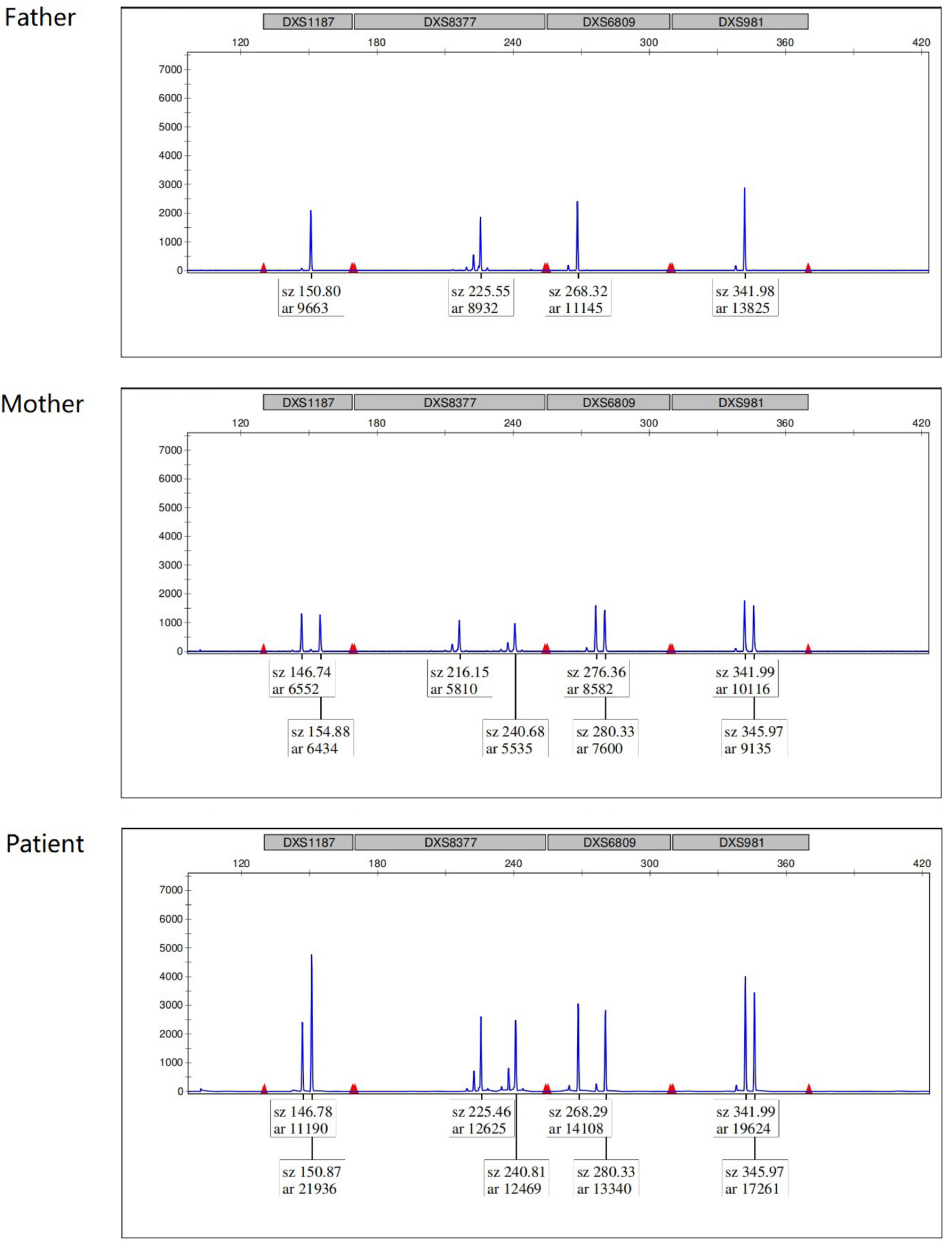
Analysis of STR loci on the X chromosome compared with her parents, the patient’s duplication locus, involved in the DXS1187 (Xq26.2), was derived from her father, and the ratio of the two fluorescent peaks in this locus was about 1:2

CMA analysis confirmed that the patient harbored a duplication of approximately 20.45 Mb at Xq23q26.3 (hg19 chrX: 114,622,584–135,068,006) (Fig. 3A). Based on the ACMG guidelines, the duplication fragment is predicted to be likely pathogenic. The duplication region encompassed 121 protein-coding genes, though none was known to be triplosensitive or disrupted by the breakpoints. No polymorphism is in the DGV database, and several similar cases have been recorded by the DECIPHER and ClinVar databases, with the manifestation of the patients including short stature, learning difficulties, mild to moderate mental retardation, and uterine anomalies. The karyotype was 46,XX,inv dup(X)(pter→q26.3::q26.3→q23::q26.3→qter) (Fig. 4). Both of her parents had a normal karyotype.

### Optical genome mapping

Optical genome mapping analysis of the DNA sample from the patient suggested that the patient has harbored two X chromosomes, with one of which containing a reverse tandem duplication (spanning approximately 19.9 Mb) at Xq23-26.3(also termed as mirror image duplication or inverted duplication) (Fig. 3B).

Analysis of the OGM result suggested that the duplication region has encompassed 107 protein-encoding genes, among which *GPC3* (OMIM 300,037), *GRIA3* (OMIM 305,915), and *STAG2* (OMIM 300,826) are associated with various diseases. However, none of the genes was known to be triplosensitive. The duplication has not been recorded in the DGV database, while a 10 Mb duplication was found in the ClinGen database (nssv13651503), associated with short stature. In the Decipher database, two cases of duplications over 16 Mb were recorded (Patients 359,237, 363,883), with the clinical phenotypes including mental retardation, intrauterine growth retardation, finger anomalies, and broad forehead.

### Parental origin of the abnormal X chromosome

The four STR loci (DXS1187 at Xq26.2, DXS8377 at Xq28, DXS6809 at Xq21.33, and DXS981 at Xq13.1) on the X chromosome were analyzed. The results showed that the patient’s duplication region involved the DXS1187 (Xq26.2) locus compared with her parents. The ratio of the two fluorescence peaks of this locus was about 1:2, and the higher peak was derived from her father, as shown in Fig. 4.

### Patterns of X chromosome inactivation in the patient

**Fig. 5:**
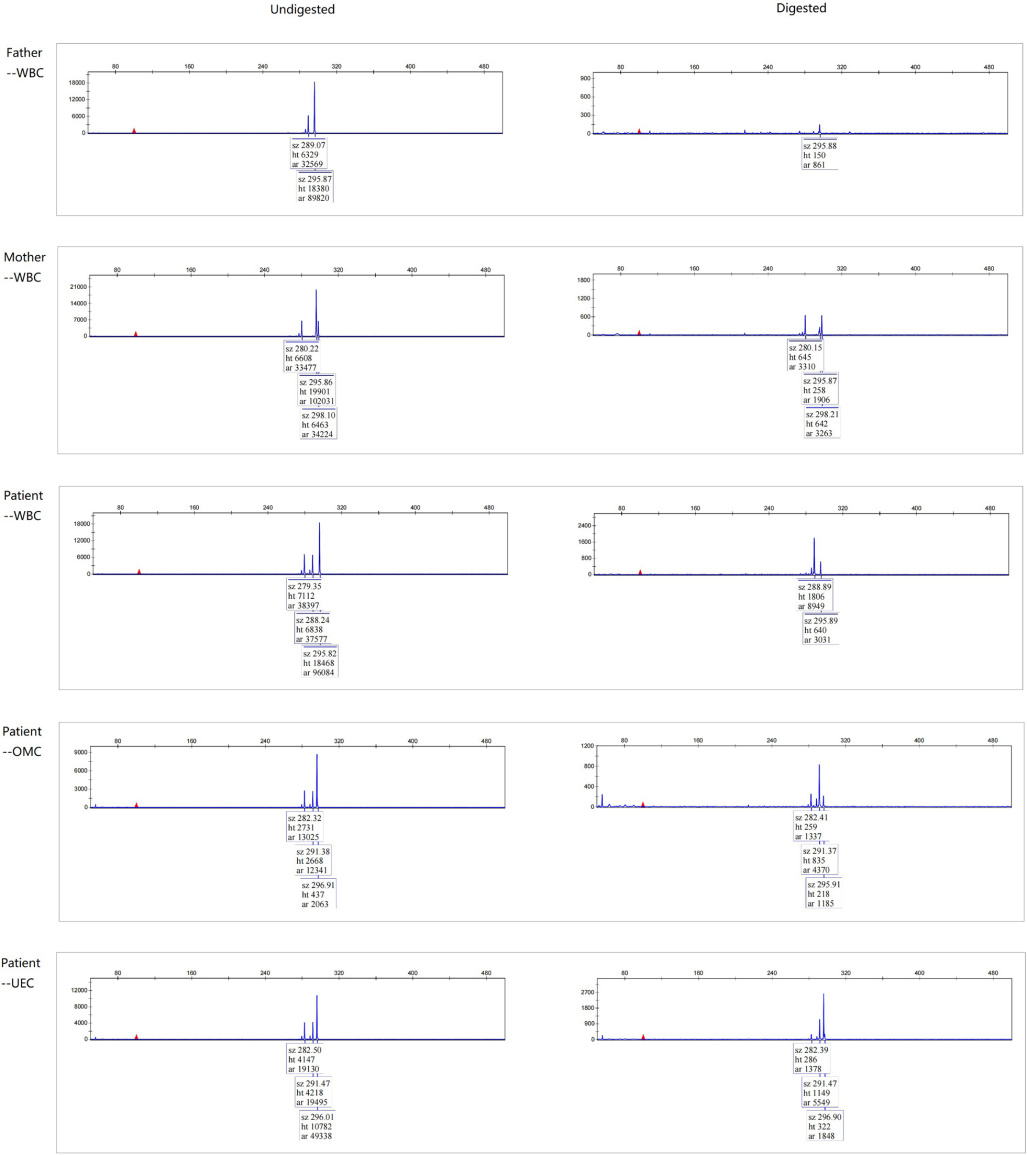
Analysis of the patterns of X chromosome inactivation the size of the fluorescence peak for the internal reference gene *B2M* was about 296 bp, and those at other positions represented specific fluorescence peaks of the *AR* gene. His father’s X chromosome was used as the control for complete digestion. The *AR* gene has a single one before digestion and disappears after digestion. Before and after digestion, the bimodal ratio of the *AR* gene on the X chromosome derived from the mother was about 50%, which suggested random inactivation. In the patient, the ratio valve of X inactivation differed with the type of tissues, e.g., 100%, 78%, and 80% for the blood, oral mucosa, and urethral epithelial cells, respectively. Compared with the STR loci of her parents, the duplicated X chromosome derived from her father was completely inactivated in the peripheral blood and partially in her oral mucosa and urethral epithelial cells. Undigested: before digestion, Digested: after digestion; WBC: white blood cells, OMC: oral epithelial cells, UEC: urethral epithelial cells

Enzymatic digestion assays were proceeded with peripheral venous blood, buccal smear, and urine samples from the patient to determine the pattern of X chromosome inactivation(XCI). As shown in Fig. 5, compared with her father (whose sole X chromosome is activated) and mother (one of her X chromosomes is randomly inactivated), the X chromosome inactivation ratio of the patient varied with the type of tissues: 100% for the blood, 78% for the oral mucosa, and 80% for the urethral epithelial cells, respectively. The comparison result of the STR loci suggested that, in all tissues, the inactivated X chromosome carried the duplicated fragment derived from her father.

### A hypothetical formation model for the inverted duplication of the paternal X chromosome

**Fig. 6:**
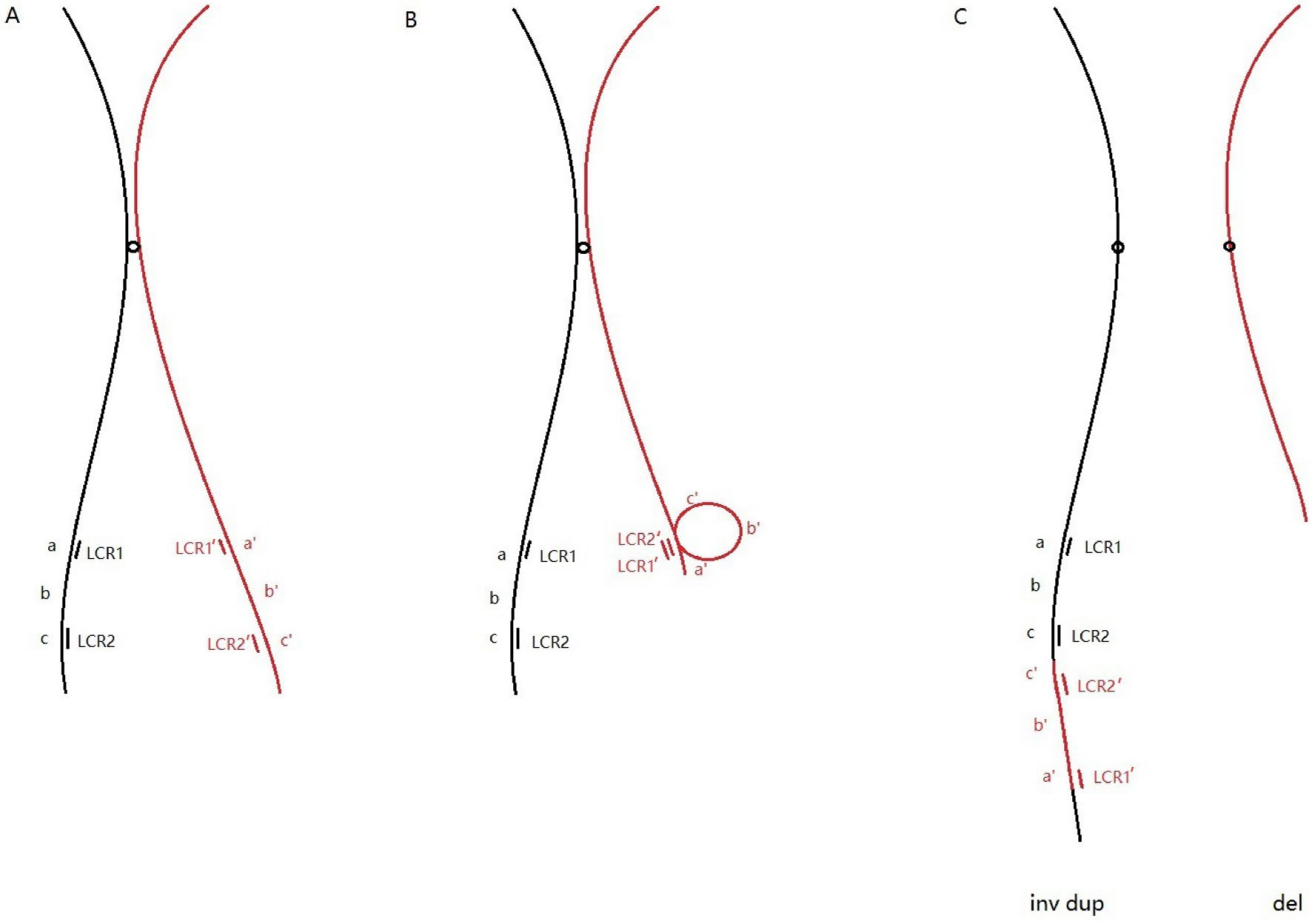
A hypothetical formation model for the inverted duplication of the paternal X chromosome during meiosis II of the paternal germ cell (**A**), LCR1’ and LCR2’ were mismatched because of the similar repeat sequences between the LCRs (**B**), resulting in unequal exchange and the production of inv-dup-type and del-type gametes (**C**), and the former is passed on to the patient

We postulate that the intrachromosomal LCRs are responsible for the inverted duplication of the X chromosome. In sperm meiosis II, the recombination of the sister chromosome results in inverted repeated sequences through the breakage and reunion of the paralogous at Xq23 and Xq26.3, as shown in Fig. 6.

### Summary of clinical phenotype in females with de novo duplications Xq23-q26

Xq23-Xq26.3 with an inverted duplication of this patient was detected by the OGM method. Compared to the duplication region with several other patients (Table 1), all share common clinical phenotypes such as growth retardation, developmental delay, and minor anomalies. However, our patient is an adult with extremely short height, and she is infertile with a unilateral horn. We have no further information on the adult height and fertility of the other three little girls in the table. Therefore, it is impossible to judge whether the inverted duplication is the cause of her extremely short stature and infertility.

**Table 1:** Summary of clinical phenotype in females with de novo duplications Xq23-q26

References	Duplicated region	Inheritance	Clinical phenotype	Methods
This study	Xq23-Xq26.3 (20 Mb), inverted duplication	De novo	A 26-year-old female with extremely short stature and mild mental deficiency. She has proportionate dwarfism with a height of 135.6 cm (< 2SD) and a weight of 32.15 kg (< 2SD). The patient has had a poor appetite and slow eating since childhood. She was irritable and bad-tempered, hypotonic, and had learning difficulties. She had deformities such as small hands, tapered fingers, right fifth finger flexion, triangular face, slight hypertelorism, thin lips, and mild micrognathia. Ultrasonography of the abdomen revealed a unilateral horn with a residual left uterine horn. She had regular but reduced menstruation. She was married for two years but had not conceived without contraception.	Karyotype; Array comparative genomic hybridisation; Short tandem repetition; X-Inactivation Assay Optical genome mapping
Garcia-Heras et al. [27]	Xq23-q26	De novo	A 3-year-old girl with growth retardation, developmental delay, and minor anomalies(Increased gap between toes 1 and 2 with 2–3 partial syndactyly. Abducted and hypoplastic thumb.).	Karyotype, FISH, X-Inactivation Assay
Armstrong et al. [28]	Xq22.3-q26	De novo	A 7-year-old girl with microcephaly. Her height is at the 10th percentile, and her hands and feet are strikingly small. She is hypotonic and delayed. Asymmetries of muscle strength and leg and foot length have been notable. She has mild unilateral ptosis. She has some features of Turner syndrome and multiple other minor anomalies. The pregnancy was complicated by intrauterine growth retardation, and she was distressed during labor. During her first year, she fed poorly and failed to thrive.	Karyotype, X-inactivation assay
Deirdre et al. [29]	Xq22.3-Xq26.1 (22.9 Mb)	De novo	A 3-year-old girl presented at 11 months of age with moderate developmental delay and slow growth. Her birth weight was 2.02 kg when elective Caesarean section at 38 weeks gestation. Postnatal weight gain was slow. Development is moderately delayed; gross motor skills are delayed. Her fine motor function is delayed. She has generalized hypotonia but was not a focal neurological deficit. She is mildly dysmorphic, with an elongated, oval face, upslanting palpebral fissures, almond-shaped eyes, epicanthic folds, and broad nasal tip. Her speech is delayed, but she is a sociable little girl.	Karyotype, Array comparative genomic hybridisation
Decipher patient: 363,883 (https://www.deciphergenomics.org/)	Xq24-Xq26.2(15.59 Mb)	Unknown	Age at last clinical assessment: less than one year. Chromosomal sex: 46,XX. Abnormal 3rd finger morphology, bilateral talipes equinovarus, broad forehead, hypertelorism, intrauterine growth retardation, single transverse palmar crease.	Unknown

## Discussion

The X chromosome is known to be involved in the determination of body stature and intelligence. Well-known examples have included Turner syndrome (45,X), Fragile X syndrome (Xq28), and individuals carrying mutations of the *SHOX* gene mapped to Xp22.1. Along with the applications of microarray chips, multiple ligation-dependent probe amplification and next-generation sequencing, a number of microdeletions and microduplications in the chrX have been discovered. The altered dosage of the involved genes has facilitated their roles in the pathogenesis of related disorders. Among these, X-linked mental retardation (XLMR) or intellectual disability (ID) is a common, clinically complex, and genetically heterogeneous disease arising from various mutations on the X chromosome, which affects 1/1000 to 1/600 males and a substantial number of females [12].

Our patient has harbored a duplication at Xq23q26.3 (hg19 chrX: 114,622,584–135,068,006), and her clinical manifestations have included extremely short stature, mild mental retardation, and primary infertility. Her duplication fragment did not overlap with the *SHOX* or *FMR1* gene nor involved the pseudoautosomal region at Xp22.33. By CMA and OGM analysis, the patient’s duplication fragment spanned 20 Mb and over 100 protein-coding genes, among which 28 are in the OMIM database. Based on a database search, none of the genes is triplosensitive, and genes at the breakpoints are not known to have a haploinsufficiency effect. No polymorphisms of such genes are in the DGV database; DECIPHER and ClinVar databases have recorded multiple cases of similar pathogenic or possibly pathogenic (mainly duplications of small fragments). The main clinical manifestations of such patients have included short stature, mental retardation, mild to moderate mental retardation, and abnormal uterus. Based on the CNV interpretation guidelines of the ClinGen/ACMG in 2019, the duplication fragment of our patient was classified as likely pathogenic.

Genomic duplication may exert an effect of dose and position [13]. The former may cause phenotypic differences for the involvement of dosage-sensitive genes, while the latter may be attributed to genes disrupted by the chromosomal breakpoints. In our patient’s duplicated region, although none of the genes is known to be triple-sensitive, some of them, e.g., *AGTR2, LAMP2, GRIA3, OCRL, GPC3, PHF6*, and *HPRT1*, have been associated with XLMR. Among them, *LAMP2, GRIA3, PHF6*, and *HPRT1* genes are associated with Danon disease (300,257), Intellectual deficiency, X-linked syndrome, Wu type (300,699), BFL syndrome (301,900), Lesch Nyhan syndrome (300,322) and other syndromes. In addition to mental retardation, such patients have short stature, deformity, and other clinical manifestations. In keeping with the previous reports, our patient height and weight are the lowest among the reported adults with Xq duplication.

The duplication in our patient did not involve the inactivation center of the X chromosome (XIST) at Xq13.2. Her skewed inactivation was found in different tissues, with the inactivation ratio for blood, oral mucosa, and urine being 100%, 78%, and 80%, respectively. Furthermore, the result of STR sites suggested the duplicated X chromosome derived from her father and inactivated, which is inconsistent with the patient’s clinical phenotype. Although XCI in the patient’s central nervous system cannot be detected, the ratio of XCI of the oral mucosa may be used to estimate the rate of X inactivation in brain tissue [14]. Therefore, a small sparing number of cells from skewed inactivation of the duplicated X chromosome of paternal origin may explain the patient’s mild mental retardation. The abnormal phenotype of dup(X) females was unpredictable because of the different inactivation of abnormal dup(X) chromosomes. This phenotypic diversity is associated with some factors, such as functional dimers of the repeated X region, inter-individual differences in X inactivation patterns, tissue-dependent X activation patterns, and incomplete inactivation of repeated X chromosome segments [15]. Non-random XCI is common in individuals carrying deleterious gene mutations or unbalanced chromosomal rearrangements [16]. Cells harboring large deletions or duplications may be disadvantageous in their growth and survival and will gradually perish. Thus, unbalanced rearrangements on the X chromosome have a milder effect on the phenotype [17]. Occasionally, skewed transmission has been noted in a small proportion of females, but extreme skewing is rare (< 1% of all cases). The skewed inactivation may influence the ultimate phenotype, regardless of whether it is of a deletion or duplication type.

Other explanations for the inconsistency between the genotypes and phenotypes include gene disruption, positional effect, complex micro-rearrangement, and different patterns or accidental associations of X inactivation in various tissues [18]. In this study, skewed X inactivation has also existed in tissues derived from her father. To what extent these might have affected the patient’s physical and mental development still await further study.

Due to their impact on survival, duplicate fragments of the X chromosome may be better tolerated by the affected individual than microdeletions. Unequal crossovers during meiosis or even mitosis can result in reciprocal microduplications and microdeletions. We have recently identified a fetus carrying a microdeletion in the Xq23 region. The fetus has a high risk for trisomy 21 in mid-gestational serological screening. An ultrasonographic scan revealed mild growth retardation, with a bi-parietal diameter and head circumstance measured at − 2.31 SD and − 2.64 SD, respectively. By amniocentesis, the fetus has a 46,X,del(X)(q23) karyotype. Further analysis with CMA and CNVseq confirmed that the fetus has a deletion spanning approximately 42.7 Mb (hg19 chrX: 99,057,340–141,763,856), which has involved 262 protein-coding genes, including 24 with a haploinsufficiency effect. No polymorphism was in the DGV database. DECIPHER and ClinVar databases had several case records featuring intrauterine growth retardation, short stature, intellectual deficiency, hearing impairment, feeding difficulty, lordosis, hypothyroidism, diabetes, and decreased muscle tone. Among the involved genes, *TIMM8A*, *PLP*, *PRPS1*, *DCX*, and *SOX3* are associated with syndromic XLMR [12]. *SOX3* is associated with hypopituitarism and growth hormone deficiency dwarfism [19, 20]. *ACSL4* (*FACL4*) and *PAK3* are associated with X-linked mental deficiency types 63 and 30 [21, 22]. Although *SIZN1*, *NXF5*, and *ARHGEF6* are non-morbid, they are also associated with XLMR [23–25].

Optical genome mapping can identify cryptic chromosomal structural variations while not by conventional methods. Therefore, it is a powerful tool for chromosomal structural aberrations, especially for complex rearrangements [26]. In this study, the OGM has detected a 19.9 Mb inverted duplication in the Xq23-q26.3 (hg19 chrX: 114,622,584–135,068,006) region, which is extremely rare. There have been a few reports of inversion duplication in autosomes, but those occurring on the X chromosome have been scarce. Our case may be explained with the triple-strand rearrangement theory proposed by Van Dyke for the formation and passage of a reverse duplication of the Xq26.3-q23 region from a father to his female offspring [13].

Probably involving the particular pathogenetic mechanisms, only several interstitial Xq deletions and duplications of large segments have been reported. Compared with previously reported females with interstitial Xq duplications of similar size [27–29] (Table 1), all share common clinical phenotypes such as intrauterine growth restriction, low birth weight, postnatal growth retardation, short stature, low intelligence, and minor anomalies. However, in contrast with three previously described females with tandem duplication, our patient had inverted duplication. She was an adult with extremely short height and infertile with a unilateral horn. We have no information on the adult height and fertility of the other three little girls. Therefore, it is impossible to judge whether the inverted duplication is the cause of her extremely short stature and infertility.

An unequal crossover of low copy repeats (LCRs) sequences is a common mechanism of microduplication and microdeletion. Similar to interstitial deletions, such duplications may also arise by unequal inversion crossing between homologous sequences during meiosis phase I or sister chromatids during meiosis II, during which the inverted insertion may translocate from one DNA strand to its complementary strand, resulting in the transfer of the inverted chromosome segment to its homologs [13, 30–33]. In addition, the LCRs are found near the breakpoints at Xp23 (hg19 chrX: 114,624,594) and Xq26.3(hg19 chrX: 134,525,997). The highly homologous sequences in such LCRs can predispose to non-allelic homologous recombination (NAHR), resulting in instability of the local region [34, 35]. In addition, the LCRs are found near the breakpoints at Xp23 (hg19 chrX: 114,624,594) and Xq26.3(hg19 chrX: 134,525,997). The highly homologous sequences in such LCRs can predispose to non-allelic homologous recombination (NAHR), resulting in instability of the local region [36, 37]. We assume that intrachromosomal recombination during paternal meiosis II between paralogous sequences at Xq23 and Xq26.3 resulted in a fertilizing rea(X) spermatozoid. Most of these rea(X) are of paternal origin. The cause may be the X chromosome in male meiosis being free to refold into itself besides the X and the Y chromosomes pair at the Xp-Yp pseudoautosomal region [38]. We assume that intrachromosomal recombination during paternal meiosis II between paralogous sequences at Xq23 and Xq26.3 resulted in a fertilizing rea(X) spermatozoid. Most of these rea(X) are of paternal origin. The cause may be the X chromosome in male meiosis being free to refold into itself besides the X and the Y chromosomes pair at the Xp-Yp pseudoautosomal region [39]. We have delineated an inverted duplication on the X chromosome in a female featuring extremely short stature, mental deficiency, and uterine anomalies. After evaluating her ovarian and uterine conditions, the patient was recommended for pre-implantation genetic testing for structural rearrangement. Our result has shed light on the pathogenesis of short stature and mental deficiency associated with the X chromosome.

In this study, we have identified a *de novo* Xq23-Xq26.3 duplication with a size of approximately 20 Mb in an adult female featuring extremely short stature and mild mental deficiency. The duplication was derived from her father, was of the inversion duplication type, and involved various degrees of skewed X chromosome inactivation. The duplication region has encompassed a number of genes, among which *ARHGEF6*, *PHF6*, *HPRT1*, and *SLC9A6* are associated with X-linked mental retardation. Correlation with her phenotypes might indicate new mechanisms by which the X chromosome may lead to short stature and mental retardation. Our findings thereby may shed more light on the phenotypic implication of functional disomy of X-chromosome genes.

## Data Availability

The datasets used and/or analysed during the current study are available from the corresponding author on reasonable request.
